# Seasonally timed treatment programs for *Ascaris lumbricoides* to increase impact—An investigation using mathematical models

**DOI:** 10.1371/journal.pntd.0006195

**Published:** 2018-01-18

**Authors:** Emma L. Davis, Leon Danon, Joaquín M. Prada, Sharmini A. Gunawardena, James E. Truscott, Johnny Vlaminck, Roy M. Anderson, Bruno Levecke, Eric R Morgan, T. Deirdre Hollingsworth

**Affiliations:** 1 Department of Mathematics, University of Warwick, Coventry, UK; 2 Data Science Institute, College of Engineering, Mathematics and Physical Sciences, University of Exeter, Exeter, UK; 3 Faculty of Medicine, University of Colombo, Colombo, Sri Lanka; 4 Department of Infectious Disease Epidemiology, School of Public Health, Imperial College London, London, UK; 5 Department of Virology, Parasitology and Immunology, Ghent University, Merelbeke, Belgium; 6 Institute for Global Food Security, School of Biological Sciences, Queen’s University, Belfast, UK; 7 School of Veterinary Science, University of Bristol, Langford, UK; 8 School of Life Sciences, University of Warwick, Coventry, UK; 9 Big Data Institute, Li Ka Shing Centre for Health Information and Discovery, University of Oxford, Oxford, UK; 10 Faculty of Health & Medical Sciences, University of Surrey, Guildford, UK; Facultad de Medicina, Universidad Nacional Autonoma de Mexico, UNITED STATES

## Abstract

There is clear empirical evidence that environmental conditions can influence *Ascaris spp.* free-living stage development and host reinfection, but the impact of these differences on human infections, and interventions to control them, is variable. A new model framework reflecting four key stages of the *A. lumbricoides* life cycle, incorporating the effects of rainfall and temperature, is used to describe the level of infection in the human population alongside the environmental egg dynamics. Using data from South Korea and Nigeria, we conclude that settings with extreme fluctuations in rainfall or temperature could exhibit strong seasonal transmission patterns that may be partially masked by the longevity of *A. lumbricoides* infections in hosts; we go on to demonstrate how seasonally timed mass drug administration (MDA) could impact the outcomes of control strategies. For the South Korean setting the results predict a comparative decrease of 74.5% in mean worm days (the number of days the average individual spend infected with worms across a 12 month period) between the best and worst MDA timings after four years of annual treatment. The model found no significant seasonal effect on MDA in the Nigerian setting due to a narrower annual temperature range and no rainfall dependence. Our results suggest that seasonal variation in egg survival and maturation could be exploited to maximise the impact of MDA in certain settings.

## Author summary

Soil-transmitted helminth infections affect 1.5 billion people worldwide and mass drug adminstration (MDA) is one of the key public health measures for reducing the burden of infection. A number of experimental studies have demonstrated links between temperature and the dynamics of *Ascaris spp.* eggs, which are exposed to the environment during maturation. Field studies in a number of countries, including Sri Lanka, Saudi Arabia and South Korea have demonstrated seasonal variation in reinfection rates; in Sri Lanka significant correlations were found between reinfection and both temperature and the number of wet days recorded. The impact of these variations on transmission, and therefore control, are unknown. Using a deterministic macroparasite model we demonstrate that seasonal changes in maturation and death rates of free-living stages, as well as transmission, could result in large seasonal swings in the infective larval population and mean worm burden in the human population. Although the seasonal effects are likely to vary between species of STH due to disparate life-cycles, this could have large knock-on consequences for timings of MDA programs and expected public health outcomes for *A. lumbricoides* infections in certain settings, indicating the need for further investigation in this area.

## Introduction

Soil-transmitted helminth infections affect approximately 1.5 billion people worldwide [1], with periodic mass deworming playing a key role in control and elimination efforts. More efficient allocation of control effort resources therefore has the potential to improve the lives of many millions of people, with studies like the DeWorm3 initiative working to determine the feasibility of interrupting transmission [2]. Ascariasis, infection of the small intestine by the parasite *Ascaris lumbricoides*, is one of the most common of these infections [1] and the life cycle of the parasite involves egg exposure to environmental conditions during larval stage development [3]. Experimental studies on *Ascaris suum* eggs, a closely-related species of ascarid, have shown that changes in temperature can affect maturation, viability and mortality [4–6]. It is likely that temperature also affects *A. lumbricoides* eggs, and that data from this and related species can be used to predict climatic effects on ascariasis [7–9].

For a related ascarid in pigs, *A. suum*, high temperatures are associated with a trade-off between faster maturation and higher mortality [5], such that an optimum temperature exists for maximum viability. This optimum temperature has been estimated for the ascarid of dogs, *Toxocara canis*, as around 25°C [10], whereas at temperatures below 10°C little or no evidence of development was recorded for either *A. suum* or *T. canis*, even after multiple months of observation [6, 10]. Rainfall is also expected to impact the life-cycle and onward transmission, but there is greater uncertainty around the magnitude and mechanism of this effect. It appears that minimal rainfall is needed to maintain soil water content above a required threshold for development of *A. suum* larvae [11]. Moisture requirements are better characterised for strongylid nematodes of livestock, for which fecal matter often already contains sufficient moisture for rainfall to not be considered a limiting factor to development, at least in temperate climates [12, 13]. There is some evidence that excess water can lead to accelerated development of ascarid larvae [11] and that survival rates are higher in environments with higher moisture [14], but it is possible that the greatest impact of rainfall on the infection cycle is through transmission. Rain is associated with greater sequestration of eggs through the soil and studies have shown that soil samples taken during rainy seasons often produce the highest yield of viable *A. lumbricoides* ova [15]. Climates which exhibit wet and dry seasons may also see changes in human behaviour that could impact transmission during these periods; for example, consumption of pickled vegetables during the late autumn to winter season has previously been suggested as a driver of reinfection in South Korea [16].

Historical field studies of ascariasis have found seasonal peaks in prevalence [17, 18] and reinfection rates [16]. One study, treating at different times of year from 1977 to 1978 across six hamlets in Kyunggi Do province, Korea, found that the highest peak in transmission occurred in early spring; a difference of 23.5% was observed between the highest and lowest reinfection rates [16]. Strong seasonal variation in reinfection rates has also been recorded in Saudi Arabia [15], with the optimal period for larval survival and transmission coinciding with cooler temperatures and a brief rainy season. A more recent field study of 477 individuals in Sri Lanka [19] found positive correlations between wet-days per month and both infection and re-infection rates.

In contrast to parasite control programmes in humans, anthelmintic treatment of livestock populations routinely takes account of seasonal variation in infection pressure. Gastrointestinal nematode infection typically peaks in summer in temperate areas [20] and during the rainy season in arid and semi-arid regions [21]. Management factors such as winter housing and concentration of birthing in spring or rainy seasons, when grass availability is highest, modify these seasonal patterns [22]. Nevertheless, effects of climatic drivers, especially temperature and rainfall, on the development and survival of infective larvae are well documented [23] and explain seasonal variation in levels of infection [24]. Models in which climate drives infection pressure are able to predict observed seasonal patterns [25–27]. Treatment generally aims to protect animals during periods of heightened risk, or to eliminate egg output in advance of conditions suitable for larval development. Thus, suppression of egg output is widely used as a management tool, and is most effective when calibrated to local climatic conditions [28, 29]. In seasonally arid regions, treatment during periods hostile for free-living parasite stages was once recommended in order to minimise reinfection; however, this favours the development of anthelmintic resistance [30]. Improved ability to predict nematode infection risk for livestock in terms of climate has led to model-driven farmer decision support tools, which are sensitive to seasonal variation in infection pressure [31, 32]. For *A. suum*, egg maturation driven by summer temperatures and prolonged survival in the winter forms the basis for recommended seasonal control strategies in pigs [33].

Despite the precedent set in the veterinary sector, the majority of public health programs have yet to adopt seasonal timing of mass drug administration (MDA) for *A. lumbricoides* control due to a lack of empirical evidence on the expected impact of such a move. Drugs are distributed through existing infrastructures, such that adjusting procedure can incur significant financial and operational costs, which the benefit of seasonal treatment would have to outweigh. However, the gain in reduced public health burden from seasonally targeted treatments could be high for certain climates, with areas that see large variations in temperature and rainfall likely to display the most pronounced differences. The key aims of this theoretical study are to propose a novel model for *A. lumbricoides* transmission that incorporates some of the seasonal elements of the system, and in doing so to demonstrate the potential impact seasonally-timed treatment could have in different climates and prevalence settings.

## Methods

### *A. lumbricoides* life cycle model

**Fig 1:**
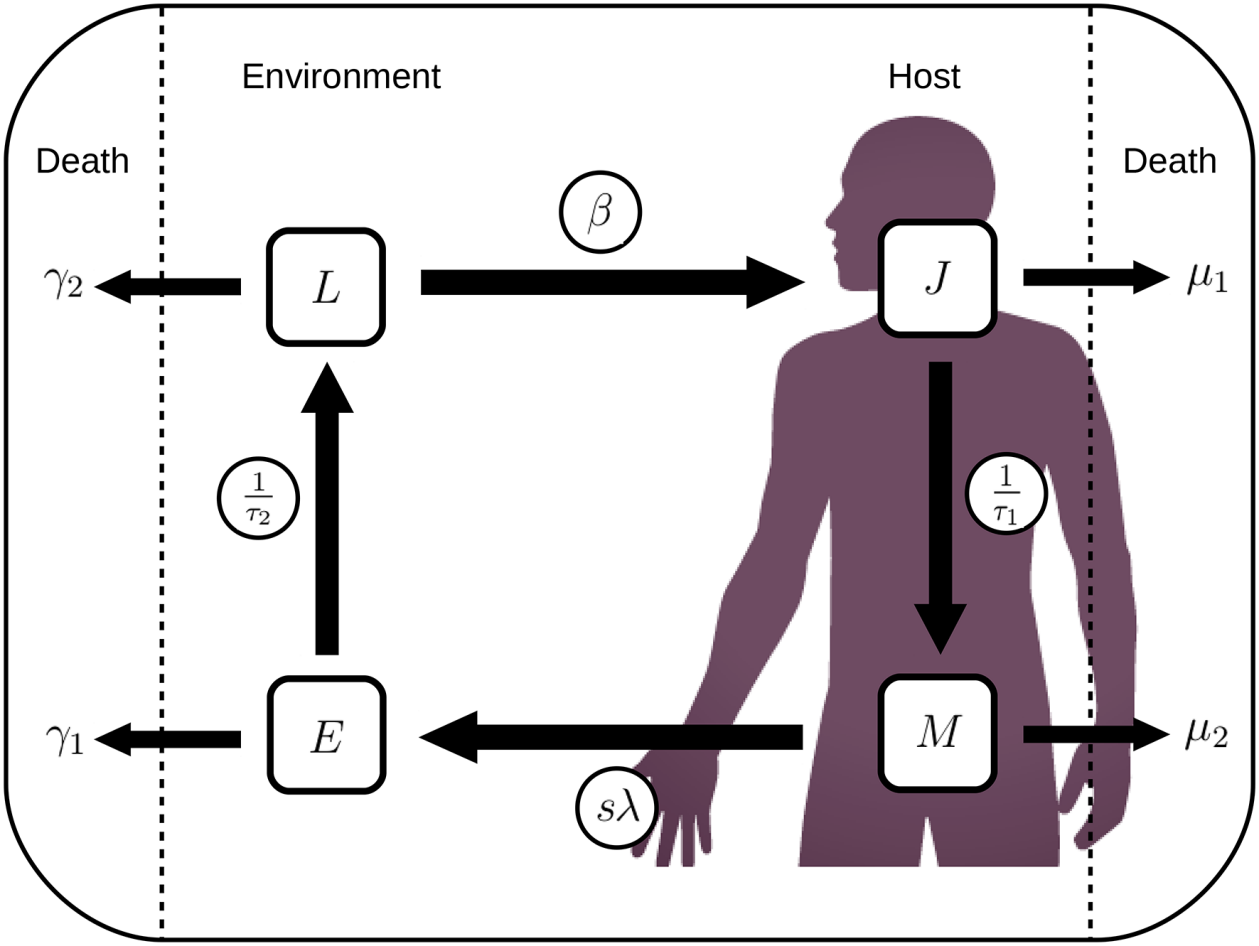
*A. lumbricoides* life cycle. Diagram depicting the model structure used to represent the *A. lumbricoides* life cycle.

A model reflecting four key stages of the *A. lumbricoides* life cycle, is used to describe the level of infection in the human population alongside the environmental egg dynamics (see Fig 1. The full equations describing the model can be found in the Supporting Information). This is a new model framework inspired by the well-established two-stage delay differential equation model developed by Anderson and May that considers the interaction between the mean worm burden (*M*) and the number of infective larval stages present in the immediate environment (*L*) [34]. Using a similar approach to Fowler et al [35], this new framework is easier to describe, implement and fit as it removes the need for delays. Here we additionally consider the mean number of juvenile worms per host (*J*) and the total count of immature eggs in the environment (*E*). This allows the removal of delays from the system such that maturation of both juvenile stages in the host and eggs in the environment can be represented as rates: 1/*τ*_1_ and 1/*τ*_2_ respectively, where *τ*_1_ and *τ*_2_ represent average maturation times.

Death rates *μ*_1_ and *μ*_2_ for the within-host stages, *J* and *M*, incorporate both parasite and host mortality. For the environmental stages, *E* and *L*, death is taken to occur at rates *γ*_1_ and *γ*_2_ respectively. The excretion rate of eggs into the environment, *sN*λ*M*, is calculated using the worm gender ratio, *s* = 0.5, the worm fecundity, λ, the human population size, *N*, and the current level of mean worm burden, *M*. Ingestion occurs at rate *βL* per host, removing eggs from the environment at rate *βNL*, where *β* represents an ingestion uptake rate.

All biological processes occurring during environmental stages are considered to be affected by seasonal factors; parameters for egg maturation time (*τ*_2_), egg mortality (*γ*_1_) and infective stage mortality (*γ*_2_) are linked to temperature through experimental data, whereas transmission (*β*) is taken to vary with rainfall. Values of model parameters are given in Table 1.

**Table 1:** Model parameter definitions and values.

Name	Definition	Value	Source
*β*	Ingestion or uptake rate	seasonal	
*τ*_1_	Maturation rate from juvenile stage to adult worm	65 (50-80)	[34]
*τ*_2_	Maturation rate from eggs to infective larvae	seasonal	[4–6]
*d*_1_	Proportion of juvenile stages that survive maturation	0.01	[34]
*d*_2_	Proportion of eggs that survive to become infective	seasonal	[5]
*μ*	Death rate of hosts (lifespan = 50 years)	5.48e-5	
*μ*_1_	Death rate of juvenile worms (including host death)	$(1-d_{1}^{1/\tau_{1}})+\mu$	n/a
*μ*_2_	Death rate of adult worms (including host death)	0.042 + *μ*	[34]
*γ*_1_	Death rate of immature eggs	$\left(\frac{1}{d_{2}}-1\right)/\tau_{2}$	
*γ*_2_	Death rate of infective larval stages	seasonal	[5]
*s*	Sex ratio in adult worms (proportion female)	0.5	[34]
λ_0_	Baseline fecundity per adult female worm	7.03 × 10^5^	[36]
*N*	Host population size	setting	

Unless specified all units are in days.

### Egg survival data

To form an evidence-base for relationships between biological model parameters and temperature we have drawn on three different experimental studies considering *A. suum* eggs. Two of these studies have been used to parameterise the average time taken for eggs to mature into infective larvae (*τ*_2_) across temperatures ranging from 5-35°C [4, 6]. The third study was used for seasonal parameterisation of the immature egg and infective larval death rates (*γ*_1_ and *γ*_2_), with a temperature range of 15-35°C [5].

The first study [4] investigated the rate of development to infectivity of a suspension of *A. suum* eggs in flasks placed inside a pig barn in Saskatchewan, western Canada. Recorded temperatures in the barn ranged from 16.8-25.5°C and increased rates of maturation were seen at higher temperatures; it took an between 21-28 days to observe development for temperatures above 23.5°C, whereas a development time of 77-84 days was recorded for a mean barn temperature of 16.8°C. This data was used as the main basis for the relationship between temperature and egg maturation time (*τ*_2_).

The second study [6] recorded the developmental stages of eggs in a coarse sand medium in an environmental chamber with 50% humidity under three temperature conditions: 5°C, 25°C and 30°C. As the humidity was maintained it can be assumed that this was not a limiting factor in development, giving temperature as the sole determinant. No development was observed at 5°C in the first month, with only marginal development being recorded across the three-month time-span of the study; no eggs reached infectivity. At 25°C and 35°C it took 19 and 17 days respectively for eggs to display successful embryonation. This data was used to extend the previous dataset to consider a wider range of temperatures in fitting *τ*_2_.

For the two external stage death rates a third study was used [5] that considered larval viability post-development and larval death rate across a temperature range of 16-34°C ± 1. Eggs were incubated in flasks containing a H_2_SO_4_ solution so moisture is also assumed to be sufficient for development and survival. Higher temperatures recorded lower viability and faster time to 90% mortality; larvae were observed as living for up to 150 days at temperatures of around 20°C, but above 25°C this quickly drops to below 50 days and above 30°C larvae survived for fewer than 10 days. The study also considered development rate, but recorded the time until 90% of the eggs had reached maturity rather than the average; this was used to provide a qualitative validation of the fitted relationship for *τ*_2_ but not considered for fitting purposes.

All seasonal egg relationships were fitted using fminsearch in Matlab 0.0.21 to minimise the squared error between the model and the data. An exponential decay curve was fitted to the maturation time data as a function of temperature; limits of function parameters give *τ*_2_ bounded below by a non-zero limit and the exponential relationship reflects the assumption that development occurs either very slowly or not at all for low temperatures [6]. The proportion of eggs successfully reaching maturity (*d*_2_) was fitted to a quartic relationship for higher temperatures and capped at a fitted maximum for lower temperatures. Immature egg death rates (*γ*_1_) were then calculated as values which would give the associated survival proportions; $\gamma_{1}=\left(\frac{1}{d_{2}}-1\right)/\tau_{2}$. Larval death rates (*γ*_2_) were derived by solving a simple differential relationship to get *γ*_2_ = −*ln*(0.1)/*m* where *m* is the time taken to achieve 90% mortality, which was fitted to an inverse tangent relationship with temperature.

### Climate data

Records of mean monthly temperature (°C) and rainfall (mm) relevant to the dates and settings we chose to investigate are taken from web archives [37, 38] and used to fit setting-specific functions. The main requirement of these functions is annual periodicity, hence a sinusoidal function provides the best approximation.

### Epidemiological data

The first dataset used to fit and validate the model originates from a field study conducted between April 1977 and September 1978 in Gyeonggi Province, South Korea [16]. The study was conducted across six hamlets (labeled A-F), each consisting of approximately 100 inhabitants, that were considered far enough apart to have independent transmission. Three rounds of biannual testing and chemotherapy were applied in each location, with intervention dates offset by a month for each hamlet to monitor different seasonal responses. The drug used was pyrantel pamoate.

The second dataset, used to investigate an alternative setting, is taken from a field study based in Osun State, Nigeria, between 2006 and 2007 [39]. The study followed two groups of 194 children, aged 12-60 months, across a period of 14 months. The treatment group received albendazole every 4 months for a year, with a follow-up assessment at 14 months; the control group received no treatment but prevalence was measured at the same intervals.

### Model implementation and fitting

The model was coded and run using Matlab R2015b, with the function ode45 used to compute numerical solutions to the differential equations. For each simulation the model was run for a 30 year period with a time step of 0.5 days to equilibrate the initial conditions before any intervention strategy was applied. Administration of anthelmintic drugs was implemented as a proportional reduction in mean worm burden; this proportion depended on efficacy, taken from the literature, and coverage, taken from the data. Treatment using albendazole was assumed to have an efficacy of 88% [40]; pyrantel pamoate is taken to have the same efficacy [40], although this is likely to be a conservative estimate [41].

In both settings transmission rate (*β*) was originally considered as an inverse tangent function of rainfall; three parameters are taken to describe the magnitude (*β*_0_), slope gradient (*a*_1_), and horizontal shift (*a*_2_) of the function. The slope gradient and shift are permitted to take a range of values to allow for either a positive or negative relationship to reflect conflicting views in the literature and as it is possible that this will change between settings due to influence from human behavioural characteristics, such as the effects of rice planting during the rainy season.

Parameters were fitted to the epidemiological datasets using approximate Bayesian computation (ABC), followed by a regression-based conditional density estimation method [42]. Uniform priors for each of the fitted parameters were defined and simulations were run using values sampled from these distributions. Simulation outputs were compared to prevalence data, filtered, and then linear regression is used to correct model inputs, resulting in a posterior distribution. Simulation outputs were filtered (keeping 1000 of 75K realisations) to maximise the binomial log-likelihood and weights were calculated using the squared errors between the model output and the data.

Model outcomes were obtained from 1000 model runs sampling parameters from the posterior distributions and credible intervals were calculated by taking the 2.5% and 97.5% quantiles of the outputs.

For the South Korean dataset villages A-C were used for fitting and then model outputs for villages D-F were compared to the other half of the data for validation. Prevalence was calculated from mean worm burden using a standard negative binomial relationship [34], with aggregation parameter *k* = 0.45 [43, 44], to capture the expected heterogeneity of infection intensity.

For the Nigerian dataset the model was fitted to the first four data points for each group and then the model predictions were compared to the fifth data point in each case. Due to results displaying a very low overall impact of rainfall on transmission the model was also fitted assuming a constant transmission rate, *β*, and Akaike Information Criterion (AIC) values were used for model selection. A negative binomial relationship was also assumed between prevalence and mean worm burden, but in this case the aggregation parameter was also fitted to the data.

## Results and discussion

### Egg survival parameters

**Fig 2:**
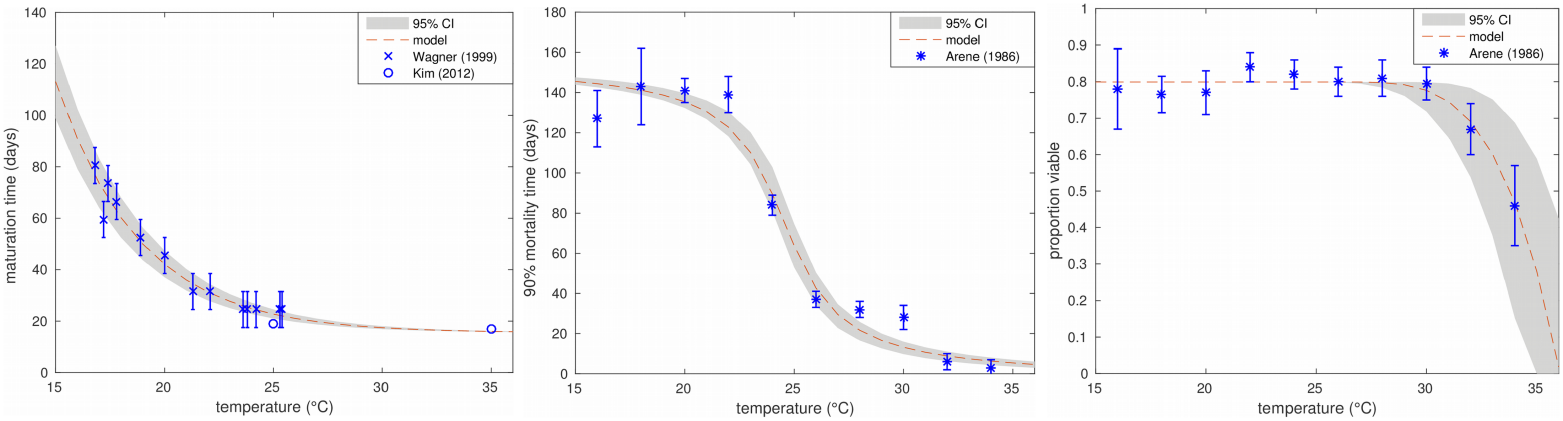
Environmental egg relationships. Fitted relationships for environmental egg parameters with temperature compared to the data. Left: Maturation time (days). Middle: Time to 90% mortality (days). Right: Proportion of eggs that develop into viable larvae.

The experimental data for all three environmental egg parameters showed strong dependence on temperature, as seen in Fig 2. The biggest effects are seen in maturation for temperatures below 20°C, for infective stage mortality above 25°C. The proportion of eggs that develop into viable larvae is mostly constant unless temperatures reach above 30°C, which is only relevant in some climates. All fitted seasonal relationships can be seen in Table 2.

**Table 2:** Fitted equations and parameters for seasonal relationships with temperature (°C), *T*.

Name	Process	Relationship	*a*_1_	*a*_2_	*a*_3_
*τ*_2_	Maturation (*E*)	*a*_1_ + *a*_2_ exp(−*a*_3_*T*)	15.5(13.0, 17.3)	4.49(3.33, 5.35) ⋅ 10^3^	0.255(0.231, 0.267)
*m*	90% mortality (*L*)	*a*_1_(arctan(*a*_2_ − *a*_3_*T*) + 1.5)	50.2(48.4, 52.3)	13.7(11.2, 16.2)	0.558(0.457, 0.660)
*d*_2_	Proportion viable	min(*a*_1_, *a*_1_ − *a*_2_(*T* − *a*_3_)^4^)	0.798(0.796, 0.801)	1.08(0.62, 1.78) ⋅ 10^−5^	26.3(25.4, 26.9)

Standard transmission models for human ascariasis would expect maturation time to be in the range of 10 to 30 days and a free living infective stage life expectancy of 28 to 84 days [34]. The fitted relationships fall in the 10-30 day range for temperatures above approximately 22.5°C, but exhibit a dramatic increase for lower temperatures. For mid-range temperatures the model predicts time to 90% mortality for infective stages to be between 40 and 120 days, which equates to a life expectancy of 17-52 days and falls within the expected range.

### Fitting and validation: Korea

The fitted parameters for transmission rate in South Korea are: *β*_0_ = 3.30(2.89, 3.82) × 10^−9^; *a*_1_ = −3.46(−3.97, −3.12); *a*_2_ = −66.8(−85.3, 56.0); such that the transmission rate $\beta =\frac{a_{1}}{\pi }\left(\arctan\left(a_{2}R+a_{3}\right)+\pi /2\right)$. This reflects an inverse relationship between rainfall and transmission, perhaps due to high rainfall resulting in infective stages being washed away from areas where uptake is likely to occur.

**Fig 3:**
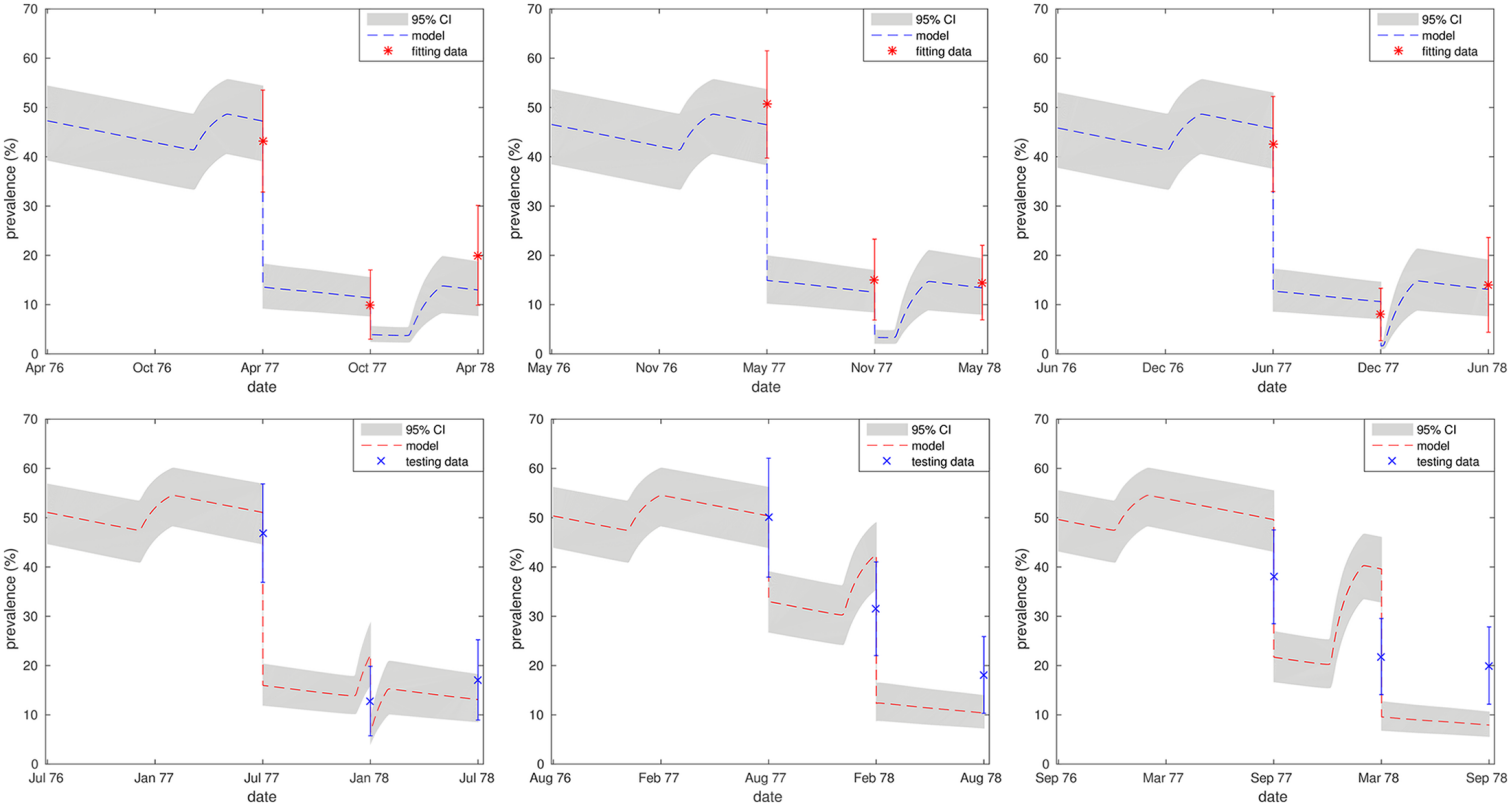
Model validation—South Korea. Fitting and testing plots for Villages A-F [16]. 95% confidence is represented by error bars on the data and 95% credible intervals by shaded regions on the model outcome. Top row (left to right): Villages A-C, fitting outcomes (*) compared with data (x). Bottom row (left to right): Villages D-F, model outcomes (- -) compared with data (x). Prevalence data was recorded pre-treatment in all cases.

Fig 3 shows both the fitting and validating outcomes for the South Korean dataset. There is excellent agreement between the model and the data used for fitting from villages A-C. Model outcomes for villages D and E are seen to provide a reasonable fit to the data when considering overlap between the respective 95% confidence intervals, with some discrepancy between the model and the data for village F.

### Fitting and validation: Nigeria

ABC results for fitting the model to the Nigerian data returned posteriors that allowed for a range of marginal positive and negative relationships between rainfall and transmission, indicating a lack of evidence to support this element of model structure, unlike the significant relationship found for the South Korean data. Comparing the Akaike Information Criterion of this model (*A*_*rain*_) to that of the reduced model (*A*_*β*_), considering constant transmission, leads us to reject the combined model with rainfall in favor of one relying only on temperature. (*A*_*rain*_ = 434.6; *A*_*β*_ = 429.6; *A*_*β*_ < *A*_*rain*_; the relative likelihood of the rainfall model is 0.082.) This implies that we do not have enough evidence to suggest rainfall is a significant predictor of disease in Nigeria. A transmission rate of *β* = 7.93*e* − 10 (7.86*e* − 10, 8.00*e* − 10) and a parasite density aggregation of *k* = 0.16 gives the best fit to the data.

**Fig 4:**
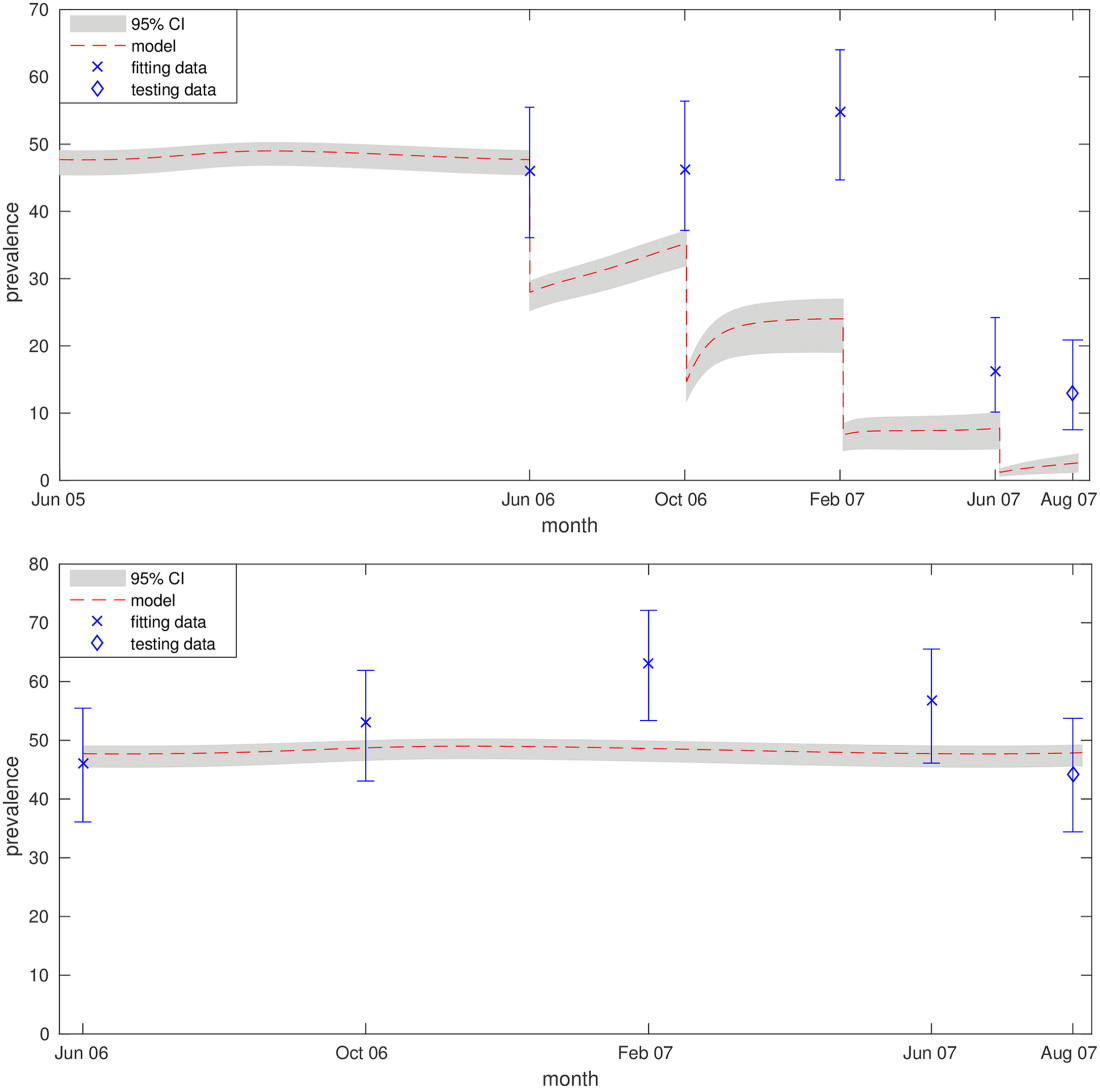
Model validation—Nigeria. Fitting plots for treatment (top) and control (bottom) branches of the 2006-07 Nigerian study [39]. 95% confidence, for the data, and 95% credible intervals, for the model, are represented with error bars and shaded regions respectively. The model (*) was fitted to the first four data points in each branch (×) and then compared to the fifth observation (◇).

Fitting outcomes for the constant transmission model capture the overall magnitude and trend of the data in both cases (see Fig 4), with reasonable agreement between the model and testing data points (August 2007). The model appears unable to capture the observed peak in cases seen across both groups during February 2007, which suggests that this increase was driven by additional factors; it is possible that sampling biases caused by behavioural change among the target population could influence such a peak.

### Impact on MDA

**Fig 5:**
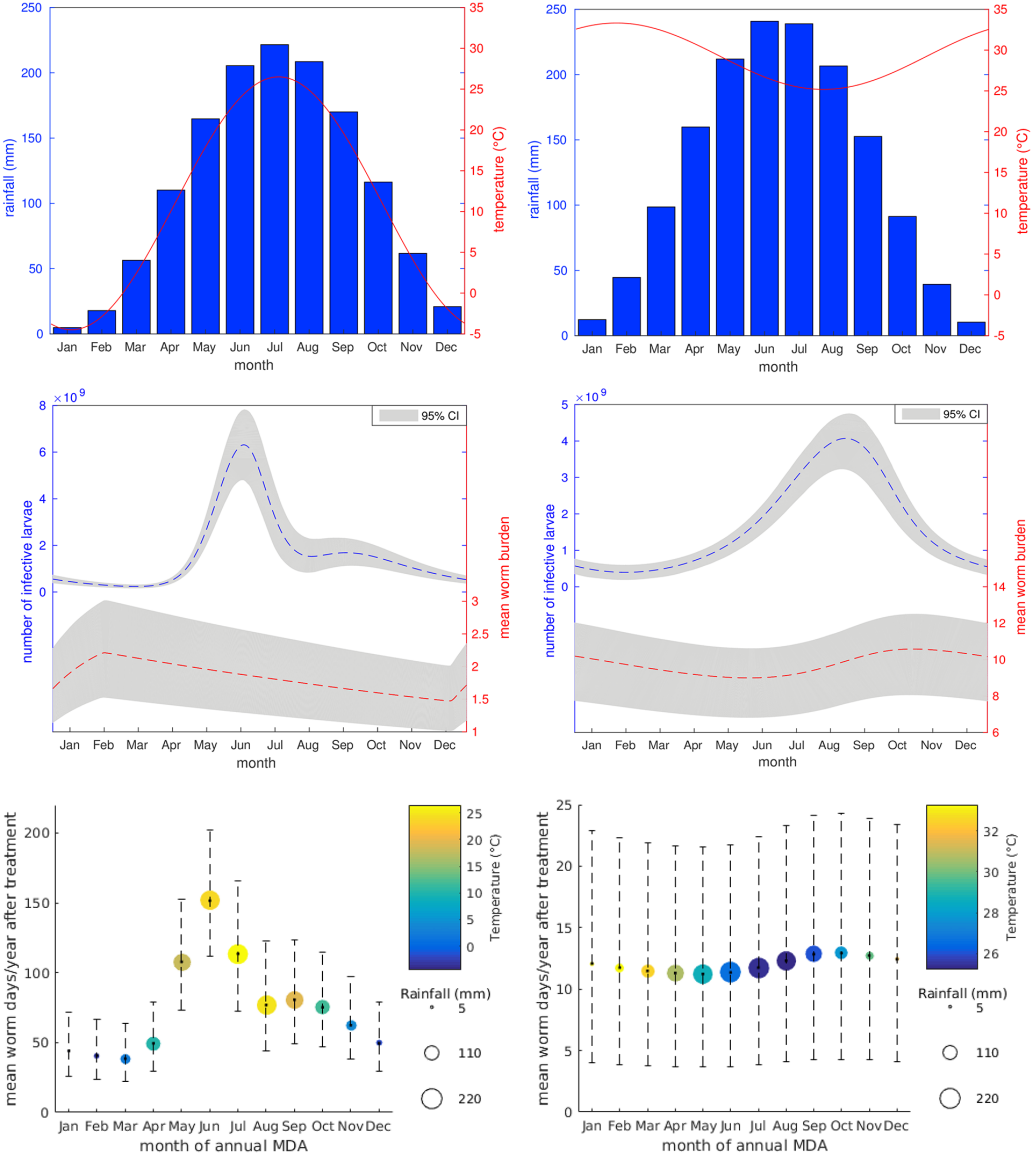
Model outcomes. Results for South Korea (left) and Nigeria (right). Top row: Fitted temperature and rainfall profiles. Middle row: Seasonal pre-control baseline profiles showing environmental levels of infective larvae and mean worm burden. Bottom row: Predicted mean number of worm days per individual across the 12 months following cessation of 4 annual MDA rounds, for treatment occurring in different months of the year. All error bands represent 95% credible intervals.

Investigating model outcomes for the South Korean setting shows that seasonal timing of MDA could result in a 74.5% difference in the number of days the average individual is infected with worms (mean worm days) across the 12 months following cessation of MDA; the best and worst case scenarios are March and June respectively. This represents a significant improvement in worm burden across the population, which could be expected to link with similar decreases in morbidity and infection intensity. Similar improvements for prevalence and levels of infectious larvae in environment are detailed in Table 3. It is also interesting to note that whilst the seasonal trend is much more noticeable in the external larval population than the mean worm burden, there is still potential for a large seasonal impact on intervention (see Fig 5).

**Table 3:** The predicted best and worst treatment months for Gyeonggi Province, South Korea, 1977.

Outcome	June (worst)	March (best)	Relative improvement
Mean worm days	151.6 (112.1—202.1)	38.6 (22.1—63.6)	74.5% (43.3—89.1%)
Prevalence	25.4% (20.9—30.3%)	9.0% (5.5—13.7%)	64.6% (34.4—81.8%)
Infectious egg count	6.26 × 10^8^ (4.48—8.51×10^8^)	1.67 × 10^8^ (0.92—2.84×10^8^)	73.3% (36.7—89.2%)

For the 12 months following cessation of 4 annual treatment rounds; mean worm days represents the total burden of infection per individual whilst other values are averaged across the time period.

In South Korea June represents a peak in levels of infectious larvae and the beginning of an uptake in transmission across the following months (see Supporting Information for estimated seasonal transmission levels), causing faster reinfection. Bringing down prevalence through MDA also results in low egg output until new adult worm infections have developed (approximately 2-3 months). As larval numbers will be naturally declining in this period it is expected that artificially reducing egg output through mass treatment will have a less marked effect on the overall population.

Contrastingly, in March, infectious larval counts are close to an annual minimum and transmission is on the decline. As the temperature picks up through April and May the larval population should experience a sharp increase, hence treating at this time is likely to limit the resulting peak and dampen future reinfection potential.

In Nigeria there is still a seasonal peak in environmental levels of infective larvae, but the model does not indicate much difference between MDA outcomes for treatment at differing times of the year (Fig 5, bottom right). This comes partially from the lack of rainfall dependence, but also due to the narrow temperature range in the region; for temperatures above 25°C both the egg maturation and larval death rates show very little variation, resulting in a reduced seasonal effect—see Supporting Information for estimated parameter values across the year.

In both cases the best time for annual treatment is predicted to occur just before the main upswing in infective larvae, the worst time coinciding with the peak. Bringing down infection levels whilst larval numbers are low starves the larval population, causing larger reductions in future infection levels; decreased transmission due to high rainfall in the summer months in South Korea exaggerates this effect.

### Conclusions

A deterministic macroparasite model has been used to investigate known relationships between temperature, rainfall and *A. lumbricoides* transmission. Model parameters were fitted to egg data from lab experiments, as well as prevalence data for two settings (South Korea 1977-78, Osun Province, Nigeria 2006-07) and used to predict the impact of these relationships on control strategies. Our results show that there could be large undetected fluctuations in the infective larval population, impacting transmission, without these effects being necessarily evident through untargeted surveys of human infection.

In South Korea fitting resulted in a negative relationship between rainfall and transmission, with the higher rainfall in the summer months causing a steep decline in transmission rate. The temperate South Korean climate is expected to provide sufficient soil moisture for year-round egg development so it is plausible that low rainfall doesn’t negatively impact the larval population. A transmission decrease due to high rainfall could be explained by the possibility eggs and larvae are being washed away through drainage systems, reducing host exposure to infection.

Osun State is located in South-Western Nigeria, where rainfall is abundant across the year; there is no dry season, as experienced by the Northern areas of the country. The lack of dependency on rainfall displayed by fitting the model to data from this region indicates that the factors influencing disease dynamics differ from those in South Korea. The infection data is not seasonally structured, and hence gives only partial information on the seasonal trends, but the peak of infection in February does imply that there could be an additional level of seasonal variation that is not captured by the model. This could be indicative of seasonal changes in population behaviour or eating habits, or other climatic factors such as humidity and soil water-content.

The model implies that optimal timing for MDA could coincide with minima in the environmental larval population, with the best treatment time predicted to be just preceding the annual upswing. These results agree with veterinary practices that advise treatment coinciding with hostile environmental conditions for the free-living stages, but we would expect a similar need for caution in this approach due to the potential for selecting for anthelmintic resistance [45]. For South Korea the much wider temperature range, as well as the inclusion of rainfall-influenced transmission in the model, led a predicted comparative decrease of 74.3% in mean worm burden between the best and worst MDA timing. In comparison, the model predicted only a 12.8% decrease for Nigeria. The climate data used was taken from as close a geographical location as possible to each study, although the monthly temperature averages used to fit these relationships will undoubtedly conceal daily fluctuations that would be expected to result in a more variable seasonal trend.

Analysis of these two contrasting settings demonstrates that the importance of seasonal factors for *A. lumbricoides* control is expected to vary dramatically between different locations, depending on local climatic and transmission patterns. It is possible that different efficacies of treatment could lead to differences in the optimal time of year for treatment, or that changing the time of year between treatments could be beneficial in some settings. Frequency and number of MDA rounds could also impact our results, but benefits from treating at a seasonally optimal time of year are expected to be cumulative.

In temperate climates, like South Korea, high ranges of temperatures may allow for significant fluctuations in larval stage development across the year and could lead to important knock-on effects for MDA programs. Although the consistent temperature pattern in Nigeria results in low predicted seasonal differences and the data presented here shows no evidence for rainfall-dependence, it is possible that rainfall could still play an important role in other settings. Although current results are subject to further evidence, we can still use the findings to gain insight into the types of settings where we might expect seasonal effects that have the potential to impact the efficacy of MDA programs.

For example, the DeWorm3 trials, which aim to test the feasibility of interrupting the transmission of STH using intensified MDA programs, are based in three countries with heterogeneous weather profiles: Benin, India and Malawi [2]. In Benin the temperature range (monthly averages of 25-30°C [46]) is narrower than that of Nigeria, so one would expect any seasonal drivers to be behavioural or rainfall-related. Temperatures are similarly high in Vellore, India, (monthly averages of 23-33°C [47]) but with a steeper drop off in the cooler months that may introduce more seasonal variation. The third setting, Malawi, exhibits a fairly narrow but much lower temperature profile (monthly averages of 17-24°C [48]) and, depending on rainfall effects, this is where we would expect seasonal MDA to have the highest impact due to the steep increase in maturation time as temperature drops under 20°C. Therefore in this setting it would be prudent to carefully consider the implications of seasonally-timed intensified MDA, as model results suggest that treatment during the cooler months could deliver maximum impact on *A. lumbricoides* transmission.

All results are subject to uncertainty, through the Bayesian fitting framework, and under the assumptions made during model construction and selection. In addition, the egg survival data used to fit the model originates from experiments on *Ascaris suum* life stages; there may still be some variation that has been unaccounted for, although previous studies have shown strong parallels between *A. suum* and *A. lumbricoides* eggs [7]. Preferred epidemiological data would include more frequent measurements, with treatment at different times of the year in parallel communities across at least four years to provide greater insight into the long term infection dynamics.

The model succeeds in qualitatively describing the biological components of the system and exhibits a good fit to both datasets, but caution must still be taken when interpreting predictions. Although the model is adapted from a well-established literature base there are still some limitations. For simplicity of calculation the helminth sex ratio within a host is not considered; infections consisting of only male or only female parasites should not result in any egg output. The assumed negative binomial relationship between mean worm burden and prevalence is also an approximation and not a true conversion. Additionally it could be worth considering the uncertainty around where transmission occurs; if infection is driven by hot-spots, such as community latrines, then these may have their own micro-climate that is less affected by the environmental conditions.

Taking seasonality into account when planning control programs can also be difficult; even in settings with clear seasonal trends there are likely to be additional complications when determining and successfully executing the optimal treatment timing. For example, the presence of other parasitic diseases in the human population could impact MDA outcomes and interfere with control measures; treatment often targets multiple STH infections and the best timing for one species may not be ideal for another. In addition, the logistics of treating at a particular time of year may be disproportionately costly or difficult for the benefits gained; it may be much easier to treat at particular times of year and moving MDA outside of these windows could result in lower coverage and hence worse overall outcomes.

If achievable, timing treatment to maxmise impact now may create future problems further down the line; veterinary experience shows that timing MDA during low periods of larval density in the environment can magnify the risk of drug resistance by imposing additional selection pressures on the system. Although anthelmintic resistance has not been definitively identified using currently available tools for human STH infections, it is still important to be cautious of any action that may encourage resistance to spread. Any seasonal recommendations for treatment timing should therefore be considered alongside the potential resistance development risk and further analysis would need to be done to inform any actions taken.

Nonetheless, our results suggest that variation in egg survival and maturation could be exploited to maximise the impact of MDA. Practically, we face the challenges of feasibility, caused by factors such as school term times and potential seasonal accessibility in hard-to-reach areas, but optimising treatment timing may be worth considering in some areas. Even though the evidence base in humans is weak there is enough grounds, combined with the depth of veterinary literature suggesting significant advantages to seasonally targeted anthelmintic therapy, to warrant further investigation.

### Supplemental methods

#### Model formulation

We consider four stages of the life cycle of *A. lumbricoides*: Juvenile worms (inside the host); Mature worms (inside the host); Eggs (developing in the environment); and Larvae (at infectious stage in the environment). These states are denoted by the letters *J*, *M*, *E* and *L* respectively; *J* and *M* are taken to be mean values per host, whereas *E* and *L* are total values in the environment.

$$\frac{dJ}{dt}=\beta L-(\frac{1}{\tau_{1}}+\mu_{1})J$$ (1)

$$\frac{dM}{dt}=\frac{1}{\tau_{1}}J-\mu_{2}M$$ (2)

$$\frac{dE}{dt}=sN\lambda M-(\frac{1}{\tau_{2}}+\gamma_{1})E$$ (3)

$$\frac{dL}{dt}=1/\tau_{2}E-\left(\beta N+\gamma_{2}\right)L$$ (4)

Parameters are as described in Table 1 and seasonal relationships are as given in Table 2. Immature egg and larval death rates were calculated from these seasonal relationships ($\gamma_{1}=\left(\frac{1}{d_{2}}-1\right)/\tau_{2}$ and *γ*_2_ = −*ln*(0.1)/*m* respectively).

For the South Korean setting transmission rate, *β*, is given by $\beta =\frac{a_{1}}{\pi }\left(\arctan\left(a_{2}R+a_{3}\right)+\pi /2\right)$. Where *β*_0_ = 3.30 × 10^−9^ (2.89, 3.82) × 10^−9^, *a*_1_ = −3.46(−3.97, −3.12) and *a*_2_ = −66.8(−85.3, 56.0). For the Nigerian setting *β* is taken as constant, *β* = 7.93 × 10^−10^ (7.86, 8.00) × 10^−10^.

Disease prevalence, *P*, in the host population is given by *P* = 1 − (1 + *M*/*k*)^−*k*^, where *k* is 0.45 for the South Korean setting (taken from a previous study) and 0.16 for the Nigerian setting (fitted to the data).

#### Model fitting

A binomial log-likelihood is used for fitting: ∑_*i*_[*x*_*i*_log(*p*_*i*_) + (*N* − *x*_*i*_)log(1 − *p*_*i*_)], where *x*_*i*_ were the positive cases from the data, *N* was the total population size and *p*_*i*_ were the model prevalences.

#### Setting-specific seasonal parameter estimation

**Fig 6:**
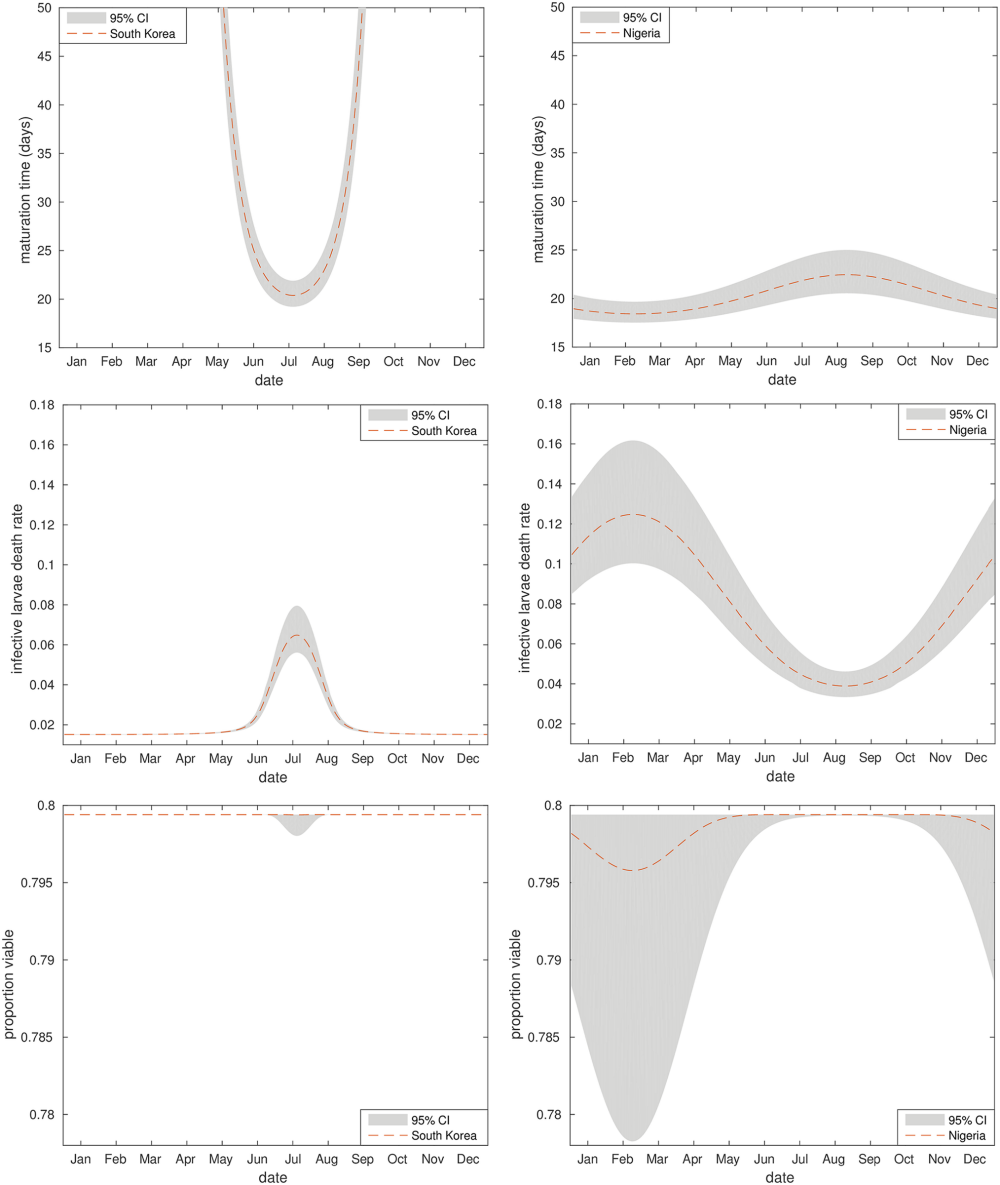
Seasonal external stage parameters. Annual parameter values for South Korea (left) and Nigeria (right). From top to bottom: maturation time (days); daily death rate of infective larval stages; proportion of eggs that are viable following maturation. Depicted as best fit model averages with 95% credible intervals.

Fig 6 shows the model estimates for setting-specific seasonal parameter values. The maturation time relationships show a key difference between the two settings; in Nigeria the high temperatures result in low-level fluctuations in maturation time around the 20 day mark, but the drop-off in temperature over the winter in South Korea is expected to produce a significant slow down in maturation across this period—with more than half the year seeing average maturation times of greater than 50 days.

In South Korea the model considers larval mortality to be very low across the entire year, due to low overall temperatures, with a peak during the summer months between May and September. In contrast, larval mortality in Nigeria is taken to fluctuate across the year, but with a higher average death rate. However, in both settings the temperatures don’t get high enough for the model to predict much effect on the proportion of eggs that remain viable following maturation.

**Fig 7:**
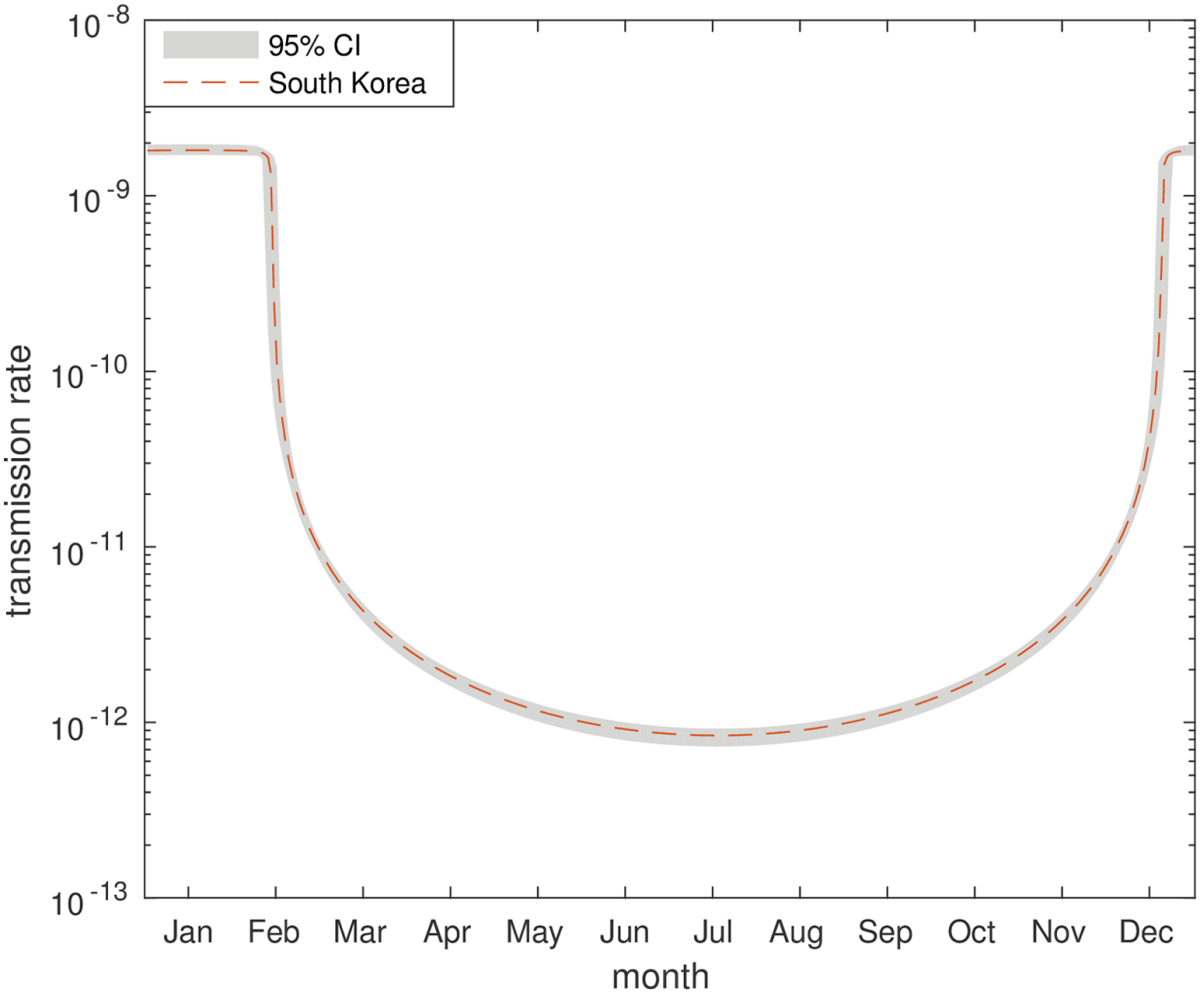
Seasonal transmission. Annual values for the transmission rate (*β*) in Osun, South Korea. Depicted as best fit model averages with 95% credible intervals.

Fig 7 shows the fitted rainfall-dependent relationship for South Korea. Transmission is at its lowest during the months that see the most rain, with a sharp increase as rainfall declines into the driest months. This could be due to heavy rain washing eggs away through drainage systems, hence reducing transmission, or human behavioural traits.

#### Additional treatment outcome plots

**Fig 8:**
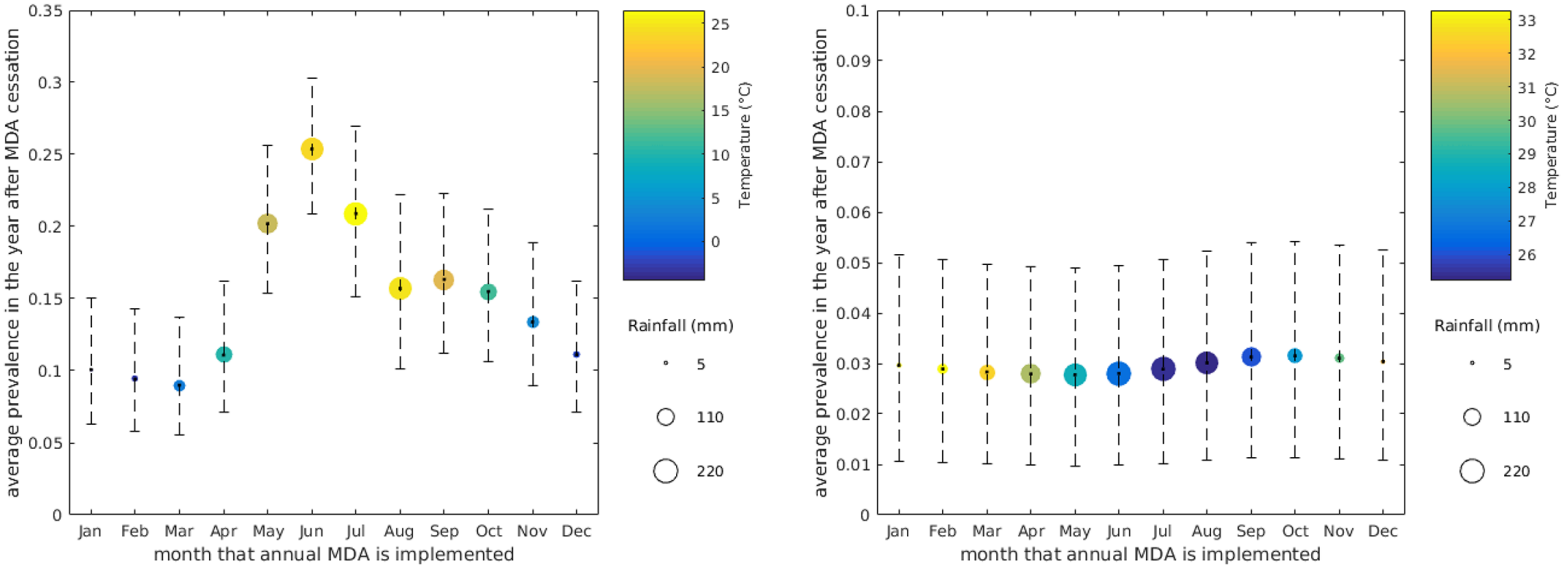
Seasonal MDA outcomes: Prevalence. Average prevalence (proportion of the population) across the 12 months following cessation of four annual MDA rounds for South Korea (left) and Nigeria (right). Depicted as best fit model averages with 95% credible intervals.

Fig 8 shows the average predicted prevalence in the 12 months immediately following 4 rounds of seasonally-timed annual MDA, with treatment times during different months of the year, for both settings. We see a very similar trend to the mean worm days plots in Fig 5, with large seasonal differences in South Korea and no evidence for any significant difference in Nigeria.
